# Mutation and Selection on the Wobble Nucleotide in tRNA Anticodons in Marine Bivalve Mitochondrial Genomes

**DOI:** 10.1371/journal.pone.0016147

**Published:** 2011-01-18

**Authors:** Hong Yu, Qi Li

**Affiliations:** Fisheries College, Ocean University of China, Qingdao, Shandong, China; Newcastle University, United Kingdom

## Abstract

**Background:**

Animal mitochondrial genomes typically encode one tRNA for each synonymous codon family, so that each tRNA anticodon essentially has to wobble to recognize two or four synonymous codons. Several factors have been hypothesized to determine the nucleotide at the wobble site of a tRNA anticodon in mitochondrial genomes, such as the codon-anticodon adaptation hypothesis, the wobble versatility hypothesis, the translation initiation and elongation conflict hypothesis, and the wobble cost hypothesis.

**Principal Findings:**

In this study, we analyzed codon usage and tRNA anticodon wobble sites of 29 marine bivalve mitochondrial genomes to evaluate features of the wobble nucleotides in tRNA anticodons. The strand-specific mutation bias favors G and T on the H strand in all the 29 marine bivalve mitochondrial genomes. A bias favoring G and T is also visible in the third codon positions of protein-coding genes and the wobble sites of anticodons, rejecting that codon usage bias drives the wobble sites of tRNA anticodons or tRNA anticodon bias drives the evolution of codon usage. Almost all codon families (98.9%) from marine bivalve mitogenomes support the wobble versatility hypothesis. There are a few interesting exceptions involving tRNA^Trp^ with an anticodon CCA fixed in Pectinoida species, tRNA^Ser^ with a GCU anticodon fixed in Mytiloida mitogenomes, and the uniform anticodon CAU of tRNA^Met^ translating the AUR codon family.

**Conclusions/Significance:**

These results demonstrate that most of the nucleotides at the wobble sites of tRNA anticodons in marine bivalve mitogenomes are determined by wobble versatility. Other factors such as the translation initiation and elongation conflict, and the cost of wobble translation may contribute to the determination of the wobble nucleotide in tRNA anticodons. The finding presented here provides valuable insights into the previous hypotheses of the wobble nucleotide in tRNA anticodons by adding some new evidence.

## Introduction

Animal mitochondrial DNA has two strands of different mutation pressure which leads to differences in base frequencies between the two strands, usually with H strand being GT-rich and L strand being CA-rich [1]. Strand-specific mutation bias has been supposed to be the main force driving and maintaining the codon usage bias [2]. Animal mitochondrial genomes typically have one tRNA for each synonymous codon families, so that each tRNA anticodon essentially has to wobble to recognize two or four synonymous codons. Several factors have been hypothesized to determine the nucleotide at the wobble site of tRNA anticodons in mitochondrial genomes. One of the traditional hypotheses is the codon-anticodon adaptation hypothesis (CAAH), which states that the codon usage bias is a determining factor, and the tRNA anticodon should coevolve with codon usage and match the most abundant codon in a synonymous codon family [3], [4]. The correlation between codon usage bias and the anticodon of tRNA has been documented in vertebrate mitochondrial genomes [2]. Another traditional hypothesis is the wobble versatility hypothesis (WVH), which argues that the nucleotide at the wobble site of tRNA anticodon should be occupied by a nucleotide which is the most versatile in wobble-pairing [5]–[7]. For example, for NNY codon families, tRNA anticodons should have G at wobble sites, because G can pair with both C and U in RNA, whereas for NNR and NNN codon families, the wobble sites should be U because of the high versatility of U in wobble-pairing [8]–[10]. The wobble versatility hypothesis is generally supported in fungal mitochondrial genomes with a few exceptions [5], [9]. Xia [10] integrated the two conventional hypotheses mentioned above and developed a new general hypothesis based on wobble cost as WCH. This hypothesis invokes that the wobble cost may reduce the decoding efficiency and accuracy, and the anticodon wobble site of tRNA should be occupied by a nucleotide with low cost of wobble pairing. WCH was tested by 36 fungal mitochondrial genomes and different costs between two kinds of U:G wobble pairs was concluded [10]. In addition, other factors such as possible suppression of stop codons and historical inertia may also contribute to the determination of the wobble nucleotides in some tRNA anticodons [9].

Up to now, the studies on the overall evolution of wobble positions of tRNA anticodons in mitochondria have been reported on vertebrates and fungi [2], [5], [9], [10]. The publications revealed that in vertebrate and fungal mitogenomes the anticodon wobble positions of tRNAs responsible for decoding NNN and NNR codons families were mostly occupied by U, whereas the majority of tRNAs decoding NNY codons possessed G at the anticodon wobble positions. The vertebrate mitochondrial data were unable to ascertain between the selection hypotheses WVH and CAAH, because both hypotheses had the same predictions for the anticodon wobble sites [2], while the fungal mitochondrial data supported WVH in most cases. Many exceptions to the above rules were also found in both vertebrate and fungal mitochondria. The most notable exception was tRNA^Met^ which had a CAU anticodon in all the vertebrate and fungal mitogenomes, violating both CAAH and WVH in most cases [2], [9], [11]. In spite of studies on the evolution of tRNA anticodons in vertebrate and fungal mitochondria, there are a few documentations of selection and mutation on tRNA anticodons in invertebrate mitochondrial genomes [12], [13]. One of the possible reasons is that the asymmetry in the distribution of the protein-coding genes is various, which hinders the generalization of the mitochondrial tRNA anticodon bias to a certain extent [2]. For example, nine protein-coding genes are collinear with the L strand and four are collinear with the H strand for shrimps, whereas 12 protein-coding genes are encoded on the H strand and only one is encoded on the L strand in sea cucumber. Moreover, the extremely variable number of tRNA genes in invertebrate mitogenomes may also limit an overall study on invertebrates. For example, the mtDNA of Chaetognatha encodes only one tRNA and Demospongiae mitogenomes encodes 2 to 27 tRNAs [14].

The class Bivalvia (Mollusca) includes both marine and freshwater species. It is notable that all marine bivalve mitochondrial genomes available in GenBank encode all the genes including protein-coding genes, tRNA and rRNA genes on the H strand, in contrast to four protein-coding genes collinear with the H strand and nine collinear with the L strand in the freshwater bivalve mitogenomes reported by now [15]. Furthermore, marine bivalve mitogenomes generally have a complete set of tRNA genes, with a few exceptions of losses or duplications of some tRNA genes. Thus, marine bivalve species should be ideal materials among invertebrates for evaluating evolutionary hypotheses on the wobble nucleotide in tRNA anticodons of mitogenomes.

We here analyzed the existing 29 mitogenomes of 25 marine bivalve species and evaluated mutation and selection on the anticodon wobble positions of tRNA genes in marine bivalve mitogenomes to provide further insights into previous hypotheses of the wobble nucleotides in tRNA anticodons.

## Results and Discussion

### Strand-asymmetrical mutation bias and codon usage bias

The AT skews for the 29 marine bivalve mitochondrial genomes are all smaller than zero, indicating the occurrence of more T than A. The GC skews are all positive, suggesting a bias against the use of C (Table 1). This result demonstrates that the strand-specific mutation bias favors G and T on the H strand in marine bivalve mitochondrial genomes sequenced by now, which is congruent with most animal mitochondrial genomes. In general, the strand-biased mutation spectrum in animal mitogenomes results in an AC-rich L strand and a GT-rich H strand. However, a minority of animal taxa including flatworms, brachiopods, echinoderms, arachnids and fishes, have been found to show a reverse strand bias [1], [16]–[20].

**Table 1:** Nucleotide biases of the total genome and protein-coding genes, and number of codon families unambiguously supporting the codon-anticodon adaptation hypothesis (N_CAAH_) and the wobble versatility hypothesis (N_WVH_) in each marine bivalve mitochondrial genome.

Species	GenBank Accession No.	Total			Protein-coding genes	N_CAAH_	N_WVH_
		GT (%)	AT skew	GC skew	3rd position GT (%)		
*Crassostrea angulata*	EU672832	57.69	−0.13	0.20	61.29	0	16
*Crassostrea ariakensis*	EU672835	57.79	−0.13	0.20	60.30	0	15
*Crassostrea gigas*	AF177226	57.70	−0.13	0.20	61.18	0	16
*Crassostrea hongkongensis*	EU672834	57.28	−0.11	0.21	59.28	0	14
*Crassostrea sikamea*	EU672833	57.85	−0.13	0.21	61.77	0	15
*Crassostrea virginica*	AY905542	57.02	−0.13	0.16	58.55	0	15
*Crassostrea nippona*	HM015198	56.61	−0.10	0.19	58.99	0	12
*Crassostrea iredalei*	FJ841967	56.82	−0.10	0.20	59.78	0	14
*Saccostrea mordax*	FJ841968	58.53	−0.15	0.21	60.22	0	14
*Ostrea denselamellosa*	HM015199	58.34	−0.15	0.19	60.39	0	16
*Sinonovacula constricta*	EU880278	63.59	−0.23	0.36	70.48	0	17
*Argopecten irradians*	EU023915	62.58	−0.25	0.31	66.40	1	17
*Mimachlamys nobilis*	FJ415225	63.75	−0.25	0.31	69.82	1	18
*Chlamys farreri*	EU715252	62.34	−0.18	0.34	67.42	1	18
*Placopecten magellanicus*	DQ088274	66.57	−0.27	0.40	79.54	2	18
*Meretrix petechialis*	EU145977	65.02	−0.26	0.39	72.73	0	17
*Meretrix meretrix*	NC_013188	64.89	−0.25	0.39	72.45	0	17
*Acanthocardia tuberculata*	DQ632743	58.85	−0.18	0.17	58.51	0	16
*Hiatella arctica*	DQ632742	59.80	−0.15	0.29	61.12	0	14
*Lucinella divaricata*	EF043342	63.44	−0.24	0.33	64.98	0	17
*Loripes lacteus*	EF043341	63.29	−0.23	0.32	66.09	0	19
*Venerupis philippinarum* F	AB065375	60.08	−0.13	0.36	60.31	0	16
*Venerupis philippinarum* M	AB065374	59.99	−0.15	0.31	62.96	0	16
*Mytilus trossulus* M	DQ198225	56.47	−0.09	0.20	54.77	0	14
*Mytilus galloprovincialis* M	AY363687	56.22	−0.08	0.20	56.24	0	14
*Mytilus edulis* M	NC_006161	58.06	−0.11	0.25	53.25	0	14
*Mytilus trossulus* F	DQ198231	58.09	−0.11	0.25	57.55	0	15
*Mytilus galloprovincialis* F	NC_006886	58.02	−0.11	0.24	57.74	0	15
*Mytilus edulis* F	AY823624	56.07	−0.08	0.19	57.98	0	15
Total						5	454

A bias favoring G and T is also visible in the third codon positions of protein-coding genes (Table 1), which is consistent with the mutation bias of the strand. In particular, NNN and NNY codon families are dominated by the T-ending codons. The result suggests that codon usage bias is maintained by strand-specific mutation bias, which has also been found in vertebrate mitogenomes [2].

### tRNA anticodon bias

Except tRNA^Met^, almost all the tRNAs have G or U at the wobble sites of anticodons. The wobble nucleotides of tRNA anticodons in marine bivalve mitochondrial genomes show a strong bias towards G and U, which is congruent with the mutation bias of the H strand. This observation seems to support the mutation hypothesis of anticodon evolution rather than the selection hypothesis of anticodon adaption described by Xia [2]. The mutation hypothesis of anticodon evolution argues that the strand-specific mutation pressure is the dominant force in shaping anticodon evolution and the anticodon wobble nucleotide bias should be in accord with the strand mutation bias. Conversely, the selection hypothesis of anticodon adaption contends that selection plays a significant role in shaping codon-anticodon adaption and codon usage bias drives the wobble sites of tRNA anticodons [2]. In other words, the tRNA anticodon should evolve to match the most abundant codon in a synonymous codon family. The vertebrate mitogenome data strongly support the selection hypothesis of anticodon adaptation, whereas the marine bivalve mitogenome data reject this hypothesis. The selection hypothesis of anticodon adaptation is also effectively ruled out in arthropod and fungal mitogenomes, where it is common that the tRNA anticodon does not match the most commonly used codon, especially for 4-fold degenerate codon families [9], [21]. However, we still can not jump to the conclusion that the strand-specific mutation pressure is the main force driving the evolution of tRNA anticodon for marine bivalve mitogenomes. This is because the evolution of the anticodon wobble site also supports the selection on anticodon versatility, given G and U are known to be more versatile in wobble-pairing than C and A.

### Cases supporting WVH

Almost all codon families (98.9%) from the 29 marine bivalve mitogenomes support WVH (Table 1), which is similar to fungal mitogenomes (94.7%) [9]. For example, for the NNY codon families, the wobble nucleotide of the tRNA anticodon is always G in the 29 mitogenomes, while the CAAH would have always predicted A at the wobble site of the anticodon. This implies that the selection at the wobble site must be very strong. In addition, almost all the tRNAs with the exception of tRNA^Met^ decoding NNR codons possess U at the wobble position of the anticodon. The majority of tRNAs decoding NNN codon families have U at the wobble site. These cases suggest that wobble versatility plays an important role in the evolution of tRNA anticodons in marine bivalve mitogenomes.

### A few cases supporting CAAH

**Figure 1:**
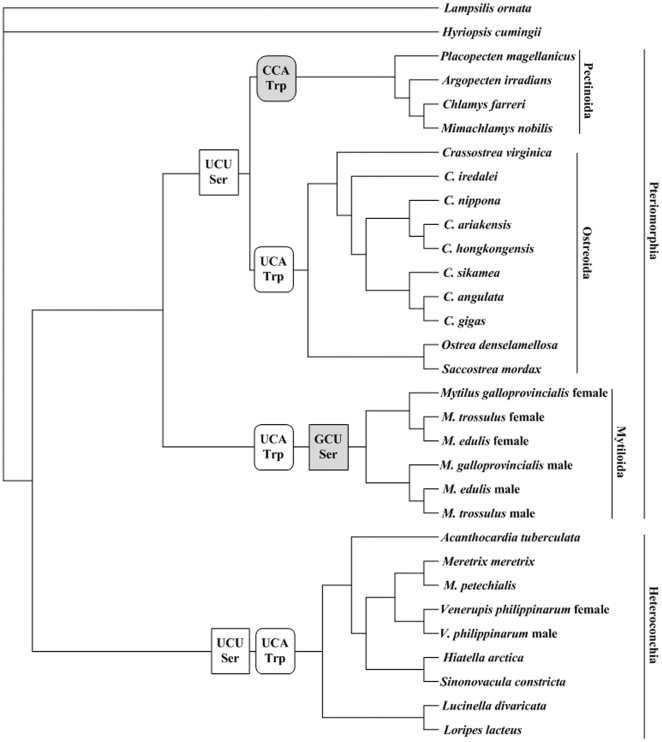
Evolution of the tRNA anticodons in marine bivalve mitochondrial genomes. The relationship presented is based on concatenated nucleotide sequences of 12 protein-coding genes by Bayesian inference analysis. Letters in gray text boxes demonstrate unusual anticodons that deviate from the usual anticodons in most marine bivalves.

A few exceptions in which CAAH is supported occur in Pectinoida mitogenomes (Table 1). Four Pectinoida species have the UGR codon family with their associated tRNA anticodons (CCA) supporting CAAH (Figure 1). The UGR codon family with its tRNA anticodon CCA consistent with CAAH was also found in some fungal mitogenomes [9]. Given UGA is a stop codon in standard genetic code and might have been captured by tRNA^Trp^ in mitochondria of an ancestral metazoan [22], the historical inertia may be a possible reason for a CCA anticodon remained for the UGR codon family in fungal mitogenomes [9]. However, in 25 other marine bivalve mitogenomes, the anticodon of tRNA^Trp^ is UCA but not CCA. Among the 25 mitogenomes with the anticodon UCA, 15 mitogenomes use more codon-UGA than codon-UGG and support both CAAH and WVH, whereas the other ten mitogenomes use more codon-UGG and support WVH. In this case, the historical inertia may not be a good probable explanation, whereas the hypothesis WCH may interpret the observation well. According to WCH, for NNR codon families, only when N_A_≪N_G_, the cost of wobble pairing for T as the wobble nucleotide (M_wT_) is larger than that for C as the wobble nucleotide (M_wC_), and WCH predicts a C at the anticodon wobble site. In other cases, M_wT_ is smaller than M_wC_ and WCH predicts a T at the anticodon wobble site [10]. The observed N_A_/N_G_ ratios in the UGR codon family range from 0.098 to 0.226 for the four Pectinoida species (Supporting Information Table S1), so M_wT_ is estimated to be larger than M_wC_ and C should be favored at the anticodon wobble site. For the 25 other mitogenomes, the N_A_/N_G_ ratios in the UGR codon family vary from 0.44 to 2.382 (Supporting Information Table S1), with the mean ratio of 1.244, much larger than those in the four Pectinoida mitogenomes. Thus, the anticodon wobble site should favor the use of T because of lower M_wT_ compared with M_wC_. That the four Pectinidae mitogenomes possess a wobble C at the tRNA^Trp^ anticodon and other 25 mitogenomes possess a wobble T confirms the prediction of WCH.

It is intriguing to find that the anticodon CCA of tRNA^Trp^ only occurs in Pectinoida species among the marine bivalves. According to the phylogenetic analysis, Ostreoida and Pectinoida are reciprocally monophyletic with Mytiloida being sister to Ostreoida+Pectinoida (Figure 1). Although Ostreoida and Pectinoida form one clade, the usage of codon UGG is different between the two orders. The usage of UGG is more frequent in the codon UGR family in Pectinoida, whereas it decreases in Ostreoida (Supporting Information Table S1). We refer that a mutation C at the wobble site of tRNA^Trp^ anticodon may occur in the common ancestor of Ostreoida and Pectinoida and was selected in Pectinoida which consequently drove the evolution of synonymous codons toward to the maximum of the codon UGG pairing with the anticodon.

The UCN codon family with the tRNA^Ser^ anticodon AGA in *P. magellanicus* mitogenome also supports CAAH, whereas all the other mitogenomes have the UGA anticodon supporting WVH. It is seldom to find an anticodon AGA of tRNA^Ser^ in other animal mitogenomes. It is therefore possible that this predicted anticodon is the result of a sequencing error, and therefore may not represent a true case supporting CAAH.

### Exceptional cases in AGN codon family

Most of the marine bivalve mitogenomes have tRNA^Ser^ with a UCU anticodon for AGN codon family supporting WVH. However, in Mytiloida mitogenomes (Figure 1), the anticodon of tRNA^Ser^ becomes GCU which does not support any available hypothesis. These cases are unlikely sequencing errors. For one thing, six mitogenomes including female-transmitted (F) genomes and male-transmitted (M) genomes of three Mytiloida species all have GCU as the anticodon. For another thing, tRNA^Ser^ with a GCU anticodon for the AGN codon family has also been found in other invertebrate mitogenomes, such as *Asterias*, *Loligo*, and some arthropod mitogenomes [12], [23], [24].

For the AGN codons, there have been a number of genetic code changes. The AGR codons, which correspond to Arg in the standard code, have been reassigned to Ser, Gly, and stop codons in different metazoan lineages [22]. Previous work suggested that the reassignment of AGR codons from Arg to Ser could have occurred in a common ancestor of all triploblastic metazoans, and the subsequent changes occurred within deuterostomes [22]. For instance, AGR codons have been reassigned to Gly in urochordates [25], and to stop codon in vertebrate mtDNA [26]. Recently, some special reassignments of AGR have also been observed. Abascal et al. [12] found parallel evolution of the genetic code in Arthropod mitogenomes, in which the AGG codon was reassigned between Lys and Ser. In addition, Temperley et al. [27] reported that human mitochondria could avoid AGA and AGG “hungry” codons by frameshifting -1 to result in a UAG stop codon, casting doubts as to whether AGR is a bona fide stop codon. Overall, the AGR codon is labile.

The molecular basis of the AGN codon family with UCU-tRNA^Ser^ can be interpreted by the high versatility of U in wobble-pairing. However, different mechanisms have been shown to address the AGN codon family with GCU-tRNA^Ser^. One of the mechanisms is anticodon base modification. Matsuyama et al. [23] proposed that the modification from G to 7-methylguanosine G (m^7^G) at the anticodon wobble position enabled the AGG codon to be decoded. Moreover, if neither a tRNA nor protein factor (i.e., release factor) competes with GCU-tRNA^Ser^ for the recognition of AGA on the ribosome, GCU-tRNA^Ser^ will recognize the AGA codon [22]. m^7^G located at the anticodon wobble position of GCU-tRNA^Ser^ has been found in *Asterias* and *Loligo* mitogenomes [23], [24]. The alternative mechanism is anticodon mutation. In arthropod mitogenomes, correlated evolution between the genetic code and the tRNA^Ser^/tRNA^Lys^ has been revealed, which invokes that the arthropod species predicted to decode AGG as Ser change the typical anticodon GCU of tRNA^Ser^ either to UCU or ACU, whereas the species predicted to decode AGG as Lys have the anticodon CUU of tRNA^Lys^ [12]. The simple mutations at the anticodons might explain the recurrence of the AGG reassignments in arthropods. In this study, the six Mytiloida mitogenomes with the GCU-tRNA^Ser^ have the anticodon UUU of tRNA^Lys^. This case is not concordant with that in arthropods and the anticodon mutation mechanism seems not to be applicable in Mytiloida mitogenomes. Thus, we speculate that modification from G to m^7^G might occur at the anticodon wobble position of tRNA^Ser^ in Mytiloida mitogenomes. In addition, modification to m^7^G also occurs at the wobble position of tRNA^Ser^ in *Asterias* and *Loligo* mitochondria. The peculiar phylogenetic distribution suggests that m^7^G may have been lost early in triploblastic metazoan diversification, and re-acquired independently in several different lineages of echinoderm and mollusk.

Mytiloida species possess an unusual system termed doubly uniparental inheritance of mtDNA (DUI) [15]. Whether the unusual anticodon GCU of tRNA^Ser^ in Mytiloida mitogenomes is related to the unusual inheritance of mtDNA? Besides Mytiloida, the marine bivalve *Venerupis philippinarum* was also found to be DUI-possessing organism [15]. However, tRNA^Ser-AGN^ is absent in *V. philippinarum* mitogenomes, so that it can not confirm the supposition. Six freshwater bivalve species have also been reported to possess DUI [15], but all the six species have the anticodon UCU for tRNA^Ser-AGN^ in both F and M mitogenomes. Hence, the anticodon GCU of tRNA^Ser^ in Mytiloida mitogenomes is not correlated to DUI.

### Special cases of tRNA^Met^ anticodon

Among the 29 marine bivalve mitogenomes, six have one tRNA^Met^ gene with the anticodon CAU, 22 have two tRNA^Met^ genes, and *P. magellanicus* has nine tRNA^Met^ genes (Table 2). Among the 22 mitogenomes with two tRNA^Met^ genes, six Mytiloida mitogenomes have a CAU-tRNA^Met^ gene and a UAU-tRNA^Met^ gene, whereas the other 16 mitogenomes have two CAU-tRNA^Met^ genes. *P. magellanicus* possesses four CAU-tRNA^Met^ genes and five UAU-tRNA^Met^ genes. It is intriguing to find variable numbers of tRNA^Met^ genes in marine bivalve mitochondrial genomes. It is commonly reported that the number of tRNA^Met^ gene in one group of species is stable. For example, all the vertebrate mitogenomes have only one tRNA^Met^ gene, while all the tunicate mitogenomes have two tRNA^Met^ genes. Two distinct genes for an initiator and an elongator tRNA^Met^, both with CAU anticodon, have been identified in the phylum Placozoa, the basal metazoan lineage [28], [29], and it has been postulated that the elongator and initiator tRNA^Met^ have been lost early in metazoan diversification, and re-acquired independently in the two distant lineages of mollusc bivalves and tunicates [14]. All the available mtDNAs of tunicates have one CAU-tRNA^Met^ gene and one UAU-tRNA^Met^ gene. However, in marine bivalve mtDNAs, single CAU-tRNA^Met^, duplicated CAU-tRNA^Met^, one CAU-tRNA^Met^ plus one UAU-tRNA^Met^, and even nine tRNA^Met^ genes are observed (Table 2). There is no distinct phylogenetic distribution of different numbers of tRNA^Met^.

**Table 2:** The effect of anticodons of tRNA^Met^ in 29 marine bivalve mitogenomes on P_UUA_ and P_AUA_.

Species	tRNA^Met^ anticodon	P_UUA_	P_AUA_	P_UUA_-P_AUA_
*Crassostrea angulata*	CAU/CAU	63.20	49.74	13.46
*Crassostrea ariakensis*	CAU/CAU	62.93	56.67	6.27
*Crassostrea gigas*	CAU/CAU	65.27	48.98	16.29
*Crassostrea hongkongensis*	CAU/CAU	71.94	64.52	7.42
*Crassostrea sikamea*	CAU/CAU	66.67	46.07	20.59
*Crassostrea virginica*	CAU/CAU	63.01	49.49	13.52
*Crassostrea nippona*	CAU/CAU	70.98	64.32	6.66
*Crassostrea iredalei*	CAU/CAU	73.26	55.43	17.82
*Saccostrea mordax*	CAU/CAU	69.04	59.46	9.58
*Ostrea denselamellosa*	CAU/CAU	66.42	49.46	16.96
*Sinonovacula constricta*	CAU	64.69	56.41	8.28
*Argopecten irradians*	CAU	50.39	37.76	12.63
*Mimachlamys nobilis*	CAU/CAU	57.62	50.61	7.01
*Chlamys farreri*	CAU/CAU	60.90	58.08	2.82
*Placopecten magellanicus*	4CAU/5UAU	37.60	33.33	4.27
*Meretrix petechialis*	CAU	56.97	54.55	2.42
*Meretrix meretrix*	CAU	58.65	54.55	4.11
*Acanthocardia tuberculata*	CAU/CAU	65.90	56.42	9.48
*Hiatella arctica*	CAU/CAU	73.96	60.73	13.23
*Lucinella divaricata*	CAU	56.60	55.62	0.99
*Loripes lacteus*	CAU	55.71	49.71	5.99
*Venerupis philippinarum* F	CAU/CAU	80.05	75.40	4.65
*Venerupis philippinarum* M	CAU/CAU	75.24	65.73	9.52
*Mytilus trossulus* M	CAU/UAU	68.24	72.44	−4.20
*Mytilus galloprovincialis* M	CAU/UAU	66.32	73.68	−7.36
*Mytilus edulis* M	CAU/UAU	69.75	69.47	0.28
*Mytilus trossulus* F	CAU/UAU	63.12	67.44	−4.32
*Mytilus galloprovincialis* F	CAU/UAU	63.70	67.62	−3.92
*Mytilus edulis* F	CAU/UAU	63.12	68.27	−5.15
Average		64.18	57.65	

The CAU-tRNA^Met^ corresponding to both AUG and AUA codons was found to have 5-formylcytidine (f^5^C) at the anticodon wobble position in bovine, nematode and squid mitochondria [30]–[32], and thus the AUA codon could be recognized. In *Drosophila* mitochondria, two kinds of CAU-tRNA^Met^ were found, one having N^6^-threonylcarbamoyladenosine (t^6^A37) at position 37 and the anticodon CAU [tRNA^Met^ (C34/t^6^A37)], and the other having A37 and the anticodon f^5^CAU [tRNA^Met^ (f^5^C34/A37)] [13]. Both of the two kinds of CAU-tRNA^Met^ can recognize AUA codon in the fruit fly mitochondrial translation system. Modification of C at the anticodon wobble position or A at the position 37 is expected to play an important role in the decoding of the AUA codon as Met [13]. Among the 29 marine bivalve mitogenomes, 22 mitogenomes have only CAU-tRNA^Met^ genes to decode both AUA and AUG codons. It is likely that tRNA^Met^ (f^5^C34/A37) or/and tRNA^Met^ (C34/t^6^A37) may exist in the 22 marine bivalve mitogenomes to stabilize the interaction between anticodon (CAU) and codon (AUA).

UAU-tRNA^Met^ can decode both AUA and AUG codons, because U can pair with both A and G. Six Mytiloida species and *P. magellanicus* mitogenomes encode both UAU-tRNA^Met^ and CAU-tRNA^Met^ genes. However, whether the mitochondrial translation systems of these seven marine bivalves acquire distinct elongator and initiator tRNA^Met^ genes needs further investigations.

Methionine is coded by both AUA and AUG codons in the 29 marine bivalve mitogenomes, with AUA more frequent than AUG in most cases, so do vertebrate and fungal mitogenomes [2], [9]. This raises the question of why the wobble C does not simply mutate to U, making the base modification unnecessary. Xia et al. [11] put forward the translation initiation and elongation conflict hypothesis to explain the unusual usage of the CAU anticodon in tRNA^Met^. This hypothesis argues that the anticodon CAU would increase the translation initiation rate but decrease the translation elongation rate, because AUG is the most efficient initiation codon, while AUA is usually more frequently used in mitogenomes. There is a conflict between translation initiation and translation elongation. According to this translation conflict hypothesis, AUA should be used relatively less frequently compared to UUA in the UUR codon family, as the anticodon CAU would impose selection against the use of AUA codon. This prediction has been confirmed in fungal mitogenomes by estimating P_AUA_ and P_UUA_ [11]. In the 29 marine bivalve mitogenomes, the mean P_AUA_ value is smaller than the mean P_UUA_ value (Table 2), in accordance with the prediction from the translation conflict hypothesis, suggesting a selection force against AUA codon. Seven mitogenomes with both CAU and UAU anticodons are predicted to favor an increased usage of AUA codon. The result confirmed this prediction. The mean (P_UUA_-P_AUA_) value is only -2.91 in the seven mitogenomes, in contrast to the other 22 mitogenomes with only CAU anticodon, where the mean (P_UUA_-P_AUA_) value is 9.53.

In conclusion, most of the nucleotides at the wobble sites of tRNA anticodons in marine bivalve mitogenomes are determined by wobble versatility. There is no evidence that the codon usage bias drives the evolution of tRNA anticodons or the tRNA anticodon bias drives the evolution of codon usage in marine bivalve mitogenomes. There are some unusual tRNA anticodons in marine bivalve mitogenomes, which may be explained by other factors such as the translation initiation and elongation conflict, and the cost of wobble translation.

## Methods

To date complete mitochondrial genomes of 23 marine bivalve species are publicly available in GenBank, of which four species (*Mytilus galloprovincialis*, *M. edulis*, *M. trossulus* and *V. philippinarum*) possess doubly uniparental inheritance of mtDNA and have F and M mitochondrial genomes. Twenty seven raw mitochondrial genomes of marine bivalve species mentioned above were downloaded from GenBank. Mitochondrial genomes for two additional marine bivalve species (*Crassostrea nippona* and *Ostrea denselamellosa*) were sequenced and added into the analyses. The information of the 29 mitochondrial genomes is shown in Table 1. All the bivalve mitochondrial genomes use genetic code 5.

The protein-coding sequences from each mitochondrial genome were extracted and codon usage quantified by using DAMBE [33]. The tRNA genes were identified by tRNAscan-SE v.1.21 [34] and DOGMA [35] using invertebrate mitochondrial genetic code and compared with the original annotations from GenBank in order to exclude incorrect annotations. Some mitochondrial genomes in GenBank were annotated incorrectly. For example, the tRNA^Ser^ gene for AGN codon family in the mitochondrial genome of *Saccostrea mordax* locates at the position 6314-6383 with an anticodon UCU, and the tRNA^Ser^ gene for UCN codon family is assigned to the position 6043-6112 with an anticodon of UGA. However, the GenBank file [FJ841968] annotated the tRNA^Ser^ gene for UCN codon family at the position 6313-6382 with an anticodon CGA and omitted tRNA^Ser^ for AGN codon family.

To investigate the nucleotide bias, skew was calculated as (A-T)/(A+T) or (G-C)/(G+C) [36]. The statistical analyses of codon usage bias were conducted according to Carullo and Xia [9]. The values of P_XUA_ were calculated according to equation as described by Xia et al. [11], in order to test whether there is tRNA^Met^-mediated selection.

Phylogenetic relationships among the marine bivalves were analyzed using concatenated nucleotide sequences from 12 protein-coding genes based on Bayesian inference (BI). Gene ATP8 was excluded from the analysis as most marine bivalve species lack this gene. Two freshwater bivalves *Hyriopsis cumingii* [FJ529186] and *Lampsilis ornate* [AY365193] were used as outgroups. The nucleotide sequences of 12 protein-coding genes were concatenated and aligned using ClustalW2 (http://www.ebi.ac.uk/Tools/clustalw2/index.html) with default parameters. Areas of dubious alignment were isolated using Gblocks [37] (default settings) and excluded from the analysis. BI analysis was conducted with MrBayes 3.1.2 [38]. Model selection was done using jMODELTEST [39]. The Akaike Information Criterion was used to determine GTR+I+G, with a gamma shape parameter of 0.63 and proportion of invariable sites of 0.039, as the most appropriate substitution model. These parameters were then used for BI phylogenetic reconstruction. The Markov Chain Monte Carlo analyses were run for 1,000,000 generations (sampling every 1000 generations) to allow adequate time for convergence. Burn-in was set to 50% leaving the last 500 sampled trees for estimating posterior probabilities.

## Supporting Information

Table Sl Usage of UGA and UGG in 29 marine bivalve mitogenomes.
(DOC)
