# Supplementary material for: Mutation and Selection on the Wobble Nucleotide in tRNA Anticodons in Marine Bivalve Mitochondrial Genomes
Source: PLoS One. 2011 Jan 18;6(1):e16147. doi: 10.1371/journal.pone.0016147 (PMC3022732; doi:10.1371/journal.pone.0016147)
Supplement: Table Sl — Usage of UGA and UGG in 29 marine bivalve mitogenomes. (DOC) [file pone.0016147.s001.doc]

| **Table S1.** Usage of UGA and UGG in 29 marine bivalve mitogenomes. | | | |
| --- | --- | --- | --- |
| **Species** | **UGA** | **UGG** | **N_A_/N_G_** |
| *Crassostrea angulata* | 75 | 37 | 2.027 |
| *Crassostrea ariakensis* | 78 | 38 | 2.053 |
| *Crassostrea gigas* | 79 | 35 | 2.257 |
| *Crassostrea hongkongensis* | 73 | 41 | 1.780 |
| *Crassostrea sikamea* | 81 | 34 | 2.382 |
| *Crassostrea virginica* | 63 | 48 | 1.313 |
| *Crassostrea nippona* | 76 | 39 | 1.949 |
| *Crassostrea iredalei* | 65 | 51 | 1.275 |
| *Saccostrea mordax* | 72 | 42 | 1.714 |
| *Ostrea denselamellosa* | 64 | 53 | 1.208 |
| *Sinonovacula constricta* | 54 | 45 | 1.200 |
| *Argopecten irradians* | 17 | 110 | 0.155 |
| *Mimachlamys nobilis* | 24 | 106 | 0.226 |
| *Chlamys farreri* | 21 | 124 | 0.169 |
| *Placopecten magellanicus* | 12 | 123 | 0.098 |
| *Meretrix petechialis* | 46 | 71 | 0.648 |
| *Meretrix meretrix* | 47 | 69 | 0.681 |
| *Acanthocardia tuberculata* | 46 | 53 | 0.868 |
| *Hiatella arctica* | 53 | 62 | 0.855 |
| *Lucinella divaricata* | 70 | 67 | 1.045 |
| *Loripes lacteus* | 40 | 91 | 0.440 |
| *Venerupis philippinarum* F | 53 | 58 | 0.914 |
| *Venerupis philippinarum* M | 47 | 59 | 0.797 |
| *Mytilus trossulus* M | 56 | 55 | 1.018 |
| *Mytilus galloprovincialis* M | 55 | 54 | 1.019 |
| *Mytilus edulis* M | 58 | 45 | 1.289 |
| *Mytilus trossulus* F | 45 | 59 | 0.763 |
| *Mytilus galloprovincialis* F | 43 | 53 | 0.811 |
| *Mytilus edulis* F | 42 | 53 | 0.792 |
